# Patient specific ankle-foot orthoses using rapid prototyping

**DOI:** 10.1186/1743-0003-8-1

**Published:** 2011-01-12

**Authors:** Constantinos Mavroidis, Richard G Ranky, Mark L Sivak, Benjamin L Patritti, Joseph DiPisa, Alyssa Caddle, Kara Gilhooly, Lauren Govoni, Seth Sivak, Michael Lancia, Robert Drillio, Paolo Bonato

**Affiliations:** 1 Department of Mechanical & Industrial Engineering, Northeastern University, 360 Huntington Avenue, Boston, MA, 02115, USA; 2 Department of Physical Medicine and Rehabilitation, Harvard Medical School, Spaulding Rehabilitation Hospital, 125 Nashua Street, Boston, MA, 02114, USA; 3 Polymesh LLC, 163 Waterman Street Providence, RI 02906-3109; 4 IAM Orthotics & Prosthetics, Inc., 400 West Cummings Park, Suite 4950, Woburn, MA, 01801, USA; 5 Harvard-MIT Division of Health Sciences and Technology, 77 Massachusetts Ave., Cambridge, MA, 02139, USA

## Abstract

**Background:**

Prefabricated orthotic devices are currently designed to fit a range of patients and therefore they do not provide individualized comfort and function. Custom-fit orthoses are superior to prefabricated orthotic devices from both of the above-mentioned standpoints. However, creating a custom-fit orthosis is a laborious and time-intensive manual process performed by skilled orthotists. Besides, adjustments made to both prefabricated and custom-fit orthoses are carried out in a qualitative manner. So both comfort and function can potentially suffer considerably. A computerized technique for fabricating patient-specific orthotic devices has the potential to provide excellent comfort and allow for changes in the standard design to meet the specific needs of each patient.

**Methods:**

In this paper, 3D laser scanning is combined with rapid prototyping to create patient-specific orthoses. A novel process was engineered to utilize patient-specific surface data of the patient anatomy as a digital input, manipulate the surface data to an optimal form using Computer Aided Design (CAD) software, and then download the digital output from the CAD software to a rapid prototyping machine for fabrication.

**Results:**

Two AFOs were rapidly prototyped to demonstrate the proposed process. Gait analysis data of a subject wearing the AFOs indicated that the rapid prototyped AFOs performed comparably to the prefabricated polypropylene design.

**Conclusions:**

The rapidly prototyped orthoses fabricated in this study provided good fit of the subject's anatomy compared to a prefabricated AFO while delivering comparable function (i.e. mechanical effect on the biomechanics of gait). The rapid fabrication capability is of interest because it has potential for decreasing fabrication time and cost especially when a replacement of the orthosis is required.

## Background

The unique advantages of rapid prototyping (RP) (also called layered manufacturing) for medical application are becoming increasingly apparent. Furthermore, developments in 3D scanning have made it possible to acquire digital models of freeform surfaces like the surface anatomy of the human body. The combination of these two technologies can provide patient-specific data input corresponding to anatomical features (via 3D scanning), as well as a means of producing a patient-specific form output (via RP). Both technologies appear to be ideally suited for the development of patient-specific medical appliances and devices such as orthoses.

This paper details a novel process that combines 3D laser scanning with RP to create patient-specific orthoses. The process was engineered to utilize surface data of the patient anatomy as a digital input, manipulate the surface data to an optimal form using Computer Aided Design (CAD) software, and then download the digital output from the CAD software to a RP machine for fabrication. The methods herein presented have the potential to ultimately provide increased freedom with geometric features, cost efficiencies and improved practice service capacity while maintaining high quality-of-service standards.

### 3D Scanning Technologies for Medical Modeling

Medical modeling is a process by which a particular part of the human body is re-created in the form of an anatomically correct digital model first and then as a physical prototype/model. Such models have had successful implementation in preoperative planning, implant design/fabrication, facial prosthetics post-surgery and teaching/concept communication to patients or medical students [1-3].

There are several 3D scanning technologies used to input the data necessary for medical modeling. Laser scanning is one method of capturing the anatomical data needed to create these models as exact replicas of the human body. 3D laser scanners use a laser beam normal to the surface to be scanned. The light reflected back from the surface is captured as a 2D projection by a CCD (charged-couple device) camera and a point cloud is created using a triangulation technique.

A second type of 3D scanner is based upon stereoscopic photogrammetry. 3D photogrammetric scanners use images captured from different points of view. Given the camera locations and orientations, lines are mathematically triangulated to produce 3D coordinates of each unobscured point in both pictures necessary to reproduce an adequate point cloud for shape and size reproduction.

Software packages that are used to create medical models for RP are unique in that they must take information from a 2D scan of the body and use that information to create a 3D model. They also have CAD functionalities to provide the possibility of optimizing the design of the model based on the application needs. The output file from the data analysis and design software is written in the standard tessellation language (STL) format, which is the most common file type used with RP machines. Once the human anatomy has been recorded and a digital model has been created, the produced STL file instructs the RP machine about how to manufacture the intended medical model [4,5].

### Rapid Prototyping for Medical Modeling and Rehabilitation

RP has been extensively used in medicine [6]. Depending on the anatomy that is being modeled and the application of interest, different types of RP machines may be most appropriate.

The most broadly used RP technique for surgical planning and training is stereolithography (SLA) [7]. An SLA machine uses a laser beam to sequentially trace the cross sectional slices of an object in a liquid photopolymer resin. The area of photopolymer that is hit by the laser partially cures into a thin sheet. The platform upon which this sheet sits is then lowered by one layer's thickness (resolution on the order of 0.05 mm) and the laser traces a new cross section on top of the first layer. These sheets continue to be built one on top of another to create the final three-dimensional shape. Some of the advantages of SLA are its high accuracy, the ability to build clear models for examination, and - with some materials - sterilization for biocompatibility.

Another RP technique known to the medical field is selective laser sintering (SLS) [e.g. 8]. This technology is similar to SLA since it relies upon a laser to sketch out the region to be built on a substrate. In this process, however, the laser binds a powder substrate rather than curing a liquid. This powder is typically rolled over the layer built before it by precision rollers, and each layer is dropped down exposing an area for a second layer to be applied. This technology can utilize stainless-steel, titanium, or nylon powders as fabrication materials.

In rehabilitation, RP has been used for the fabrication of prosthetic sockets [9,10]. It has been also proposed as a way to optimize the design of customized rehabilitation tools [11]. Research on the development of custom-fit orthoses using RP has been very limited. A 3D scanner in conjunction with SLS was used by Milusheva et al. [12,13] to develop 3D models of customized AFO's. However, the SLS prototype of the customized AFO was used only for design evaluation purposes and not as the functional prototype. Another customized AFO manufactured using SLS was presented by Faustini et al. [14]. The geometry of these AFOs was captured by Computed-Tomography (CT) scanning of an AFO built using a conventional technique rather than generating the surface model directly from the subject's anatomy.

It is clear that although some important pioneering research has already been performed in the area of RP patient-specific orthoses, several aspects of the implementation of the technique to manufacture AFOs using RP need to be addressed including: a) demonstrating the full design/manufacturing cycle starting from obtaining scans of the human anatomy to fabricating the customized orthosis; and b) performing gait analysis experiments to evaluate the mechanical effect of orthoses manufactured using RP and compare their performance with that achieved using orthoses fabricated by means of conventional techniques.

### Current Methodology to Develop Custom-Fit AFOs

**Figure 1:**
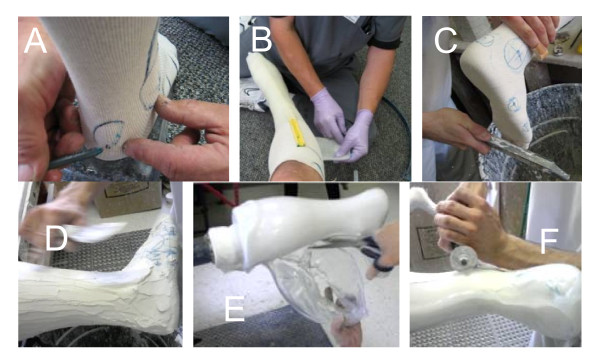
**Traditional fabrication process of an ankle foot orthosis for a patient**.

Creating a custom-fit AFO is a laborious and time-intensive manual process performed by skilled orthotists. This process is depicted in Figure 1 and can take up to 4 hours of fabrication time per unit for an experienced technician. Once the orthotist has determined the configuration and orientation of the subject's anatomy for corrective measures, the form is captured by wrapping a sock and casting the leg (Figure 1a). Markings are drawn at key locations onto the sock surface which instruct technicians later on about where to perform corrective modifications. Once the cast has set (Figure 1b), it is cut away along the anterior contour, in line with the tibia (Figure 1c). The open edge of the cast is filled and plaster is poured into the leg cavity. Starting at the heel, key surfaces are built outwards with plaster by embedding staples corresponding to surface markers (Figure 1d). Once the leg bust has been modified, pre-heated thermoplastic is vacuum formed around the plaster (Figure 1e). Once cool, the unwanted plastic is cut away, leaving an uneven 1/4" deep gash in the modified leg bust, and requiring edges on the AFO to be ground down & smoothed (Figure 1f). The back vertical surface of the removed AFO is loaded and bent forward by the technician to check for even splay during weight bearing. Should the need arise to re-fabricate a patient's AFO, the gash in the bust must be repaired before thermoforming can take place. Due to warehousing considerations, most leg busts in clinics are not kept for more than typically 2 months, so for each patient refitting (typically occurring every other year), the whole process must start from the beginning.

## Methods

**Figure 2:**
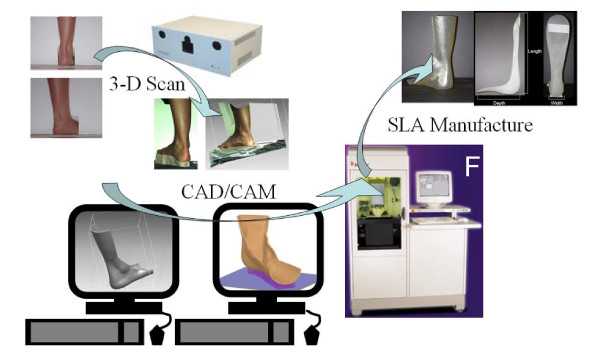
**Process used to fabricate the proof of concept AFOs**.

The main steps of the proposed method are: a) positioning the patient in a way that is suitable for scanning and taking the scan using a 3D scanner that is capable of creating a full 3D point cloud of the ankle-foot complex (or any other joint of interest); b) processing and manipulating the data from the scan to create the computer model of the desired orthosis including performing design modifications to optimize the shape of the orthosis according to the clinical needs; c) fabricating the custom-fit orthosis using a RP machine. Figure 2 illustrates the process.

To show that the proposed technique can lead to manufacturing an AFO comparable to a prefabricated one, we chose a posterior leaf spring AFO (Type C-90 Superior Posterior Leaf Spring, AliMed, Inc., Dedham, MA) as an exemplary orthotic device to be matched by using the proposed RP-based technique [15]. The RP implementation of the posterior leaf spring AFO used a 3D FaceCam 500 from Technest Inc. [16] for acquiring the data of the human's anatomy and a Viper Si2 SLA machine from 3D Systems Inc. for layered manufacturing [17].

### 3D Scanning

**Figure 3:**
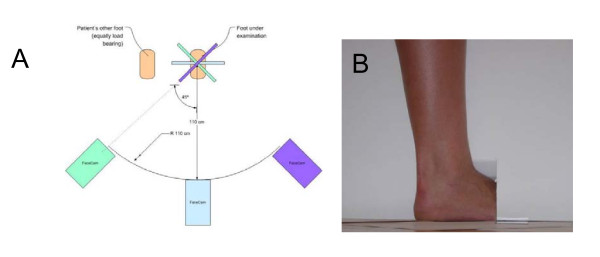
**Positioning of the foot during laser scanning**. (A) Schematic of the setup and procedure used to scan the ankle of the subject. Note the relative positions of the cameras. (B) Lateral aspect of the foot and ankle as seen from the perspective of the right camera of the scanner.

The 3D FaceCam 500 scanner from Technest Inc captures three images (two for surface shape, one for color) with a resolution of 640 × 480 pixels. During a scan, a pattern of colored light is projected onto the target surface. The reflected light from this pattern is captured by camera lenses at two different locations, which will later be used to reconstruct the shape digitally. In order to get the most accurate data possible from the 3D scans, a procedure was developed for scanning a subject's ankle and foot. The design required data from below the knee and to the posterior of the leg and also the ventral side of the foot. The camera locations for scans are dictated by its range and field of view, which directly impact the quality of the data. The scanning operation was broken down into 3 vertical images of the ankle region and 3 images of the bottom of the foot whilst the subject was not load bearing. A white background was placed around the leg to differentiate the subject's leg from extraneous data. Figure 3 shows the position of the camera for each of the scans of the ankle-foot complex while load bearing and one view of the subject's foot and ankle as seen by the 3D scanner. The non-load bearing scans were taken with the knee at about 90 deg and the shank in a vertical position.

### Software

**Figure 4:**
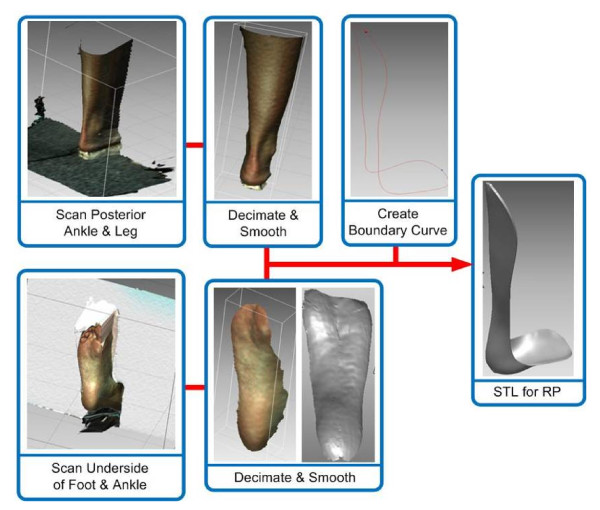
**Flow diagram of the post-scanning software procedures**.

The acquired scans were post-processed using the software *Rapidform *[18]. This software was used to clean and convert the scans by removal of unwanted points and meshing of the point cloud into a single shell. Figure 4 illustrates this process.

The process began with removing redundant data points (Figure 4). This includes data from the parts of the leg that were not needed as well as mismatching surfaces and data from the floor or background for each captured view. The points within each cloud were then connected to each other with three-sided polygons to create a surface mesh. The individual surface meshes were aligned and merged to create one complete surface model of the ankle-foot complex. The polygon surface curvature was smoothened and edges then trimmed with a boundary curve. This surface was then offset to prevent the fabricated AFO from over-compressing the subject's leg. The offset surface was extruded to a thickness of 3 mm as typically done for fitting of standard AFOs [15]. Once completed, the model was exported from *Rapidform *as a STL file.

### Rapid Prototyping

The model was manufactured using the 3D Systems Viper Si2 SLA machine [17]. This system uses a solid state Nd YVO_4 _laser to cure a liquid resin. STL files were prepared with *3D Lightyear *for part and platform settings, and *Buildstation *to optimize the machine's configuration.

The effectiveness of using RP for the application at hand is largely dependent on material properties. The prefabricated AFO selected for the study (i.e. the one we attempted to match using the proposed methodology based on RP) was the Type C-90 Superior Posterior Leaf Spring from AliMed [15]. This AFO comes in a pre-determined range of sizes of injection molded polypropylene.

Two different AFOs, each fabricated with a different material, were built using the Viper SLA machine. The first material was the Accura 40 resin that produced a rigid AFO while the second AFO was more flexible as it was manufactured using the DSM Somos 9120 Epoxy Photopolymer. This resin is biocompatible for superficial exposure and offers good fatigue properties relative to the polypropylene [19]. Material properties are compared in Table 1.

**Table 1:** AFO material properties:

Description	Unfilled Polypropylene	Accura SI 40	Somos^® ^9120 UV
Tensile Strength (MPa)	31 - 37.2	57.2 - 58.7	30 -32
Elongation (%)	7 - 13	4.8 - 5.1	15 - 25%
Young's Modulus (GPa)	1.1 - 1.5	2.6 - 3.3	1.2 - 1.4
Flexural Strength (MPa)	41 - 55	93.4 - 96.1	41 - 46
Flexural Modulus (MPa)	1172 - 1724	2836 - 3044	1310 - 1455

a) polypropylene used with the standard, prefabricated AFO); b) Accura SI 40 used with the rigid RP AFO and c) the epoxy photopolymer Somos 9120 used with the flexible RP AFO.

### Gait Analysis

Gait studies were conducted at Spaulding Rehabilitation Hospital, Boston, MA using a motion capture system. We collected data from a healthy subject (the one for which scans were taken in order to manufacture the AFO) walking without an AFO, walking with the above-mentioned standard, prefabricated AFO, and walking with each of the AFOs manufactured using the proposed RP-based technique. The subject wore the AFOs on the right side. Four different conditions were therefore tested: 1) with sneakers and no AFO (No AFO); 2) with the standard, prefabricated polypropylene AFO (Standard AFO); 3) with the rigid AFO made with the Accura 40 resin (Rigid RP AFO), and 4) with the flexible AFO made from the Somos 9120 resin (Flexible RP AFO).

**Figure 5:**
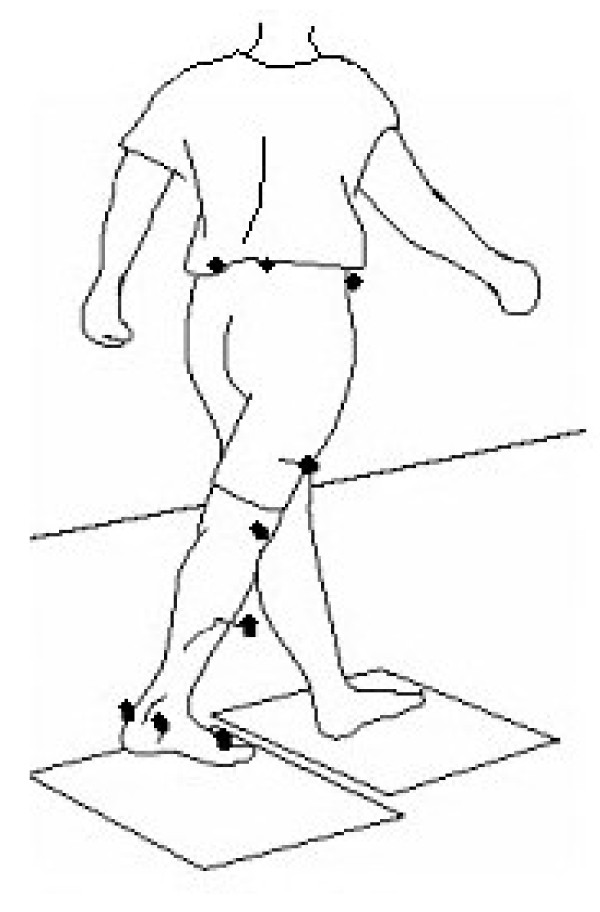
**Position of the reflective markers used during the gait analyses**.

Reflective markers were placed on the following anatomical landmarks: bilateral anterior superior iliac spines, posterior superior iliac spines, lateral femoral condyles, lateral malleoli, second metatarsal heads, and the calcanei (Figure 5). Additional markers were also rigidly attached to wands and placed over the mid-femur and mid-shank. The subject was instructed to ambulate along a walkway at a comfortable speed for all of the walking trials. An 8-camera motion capture system (Vicon 512, Vicon Peak, Oxford, UK) recorded the three-dimensional trajectories of the reflective markers during the walking trials. Two force platforms (AMTI OR6-7, AMTI, Watertown, MA) embedded in the walkway recorded the ground reaction forces and moments. Data was gathered at 120 Hz. Ten walking trials with foot contacts of each foot onto the force platforms were collected for each testing condition.

Gait parameters derived from the walking trials included spatio-temporal parameters and kinematics and kinetics of the hip, knee and ankle of each leg in the sagittal plane. Kinematics (joint angles) and kinetics (joint moments and powers) were estimated using a standard model (Vicon Plug-in-Gait, Vicon Peak, Oxford, UK).

## Results

### AFO Fabrication

**Figure 6:**
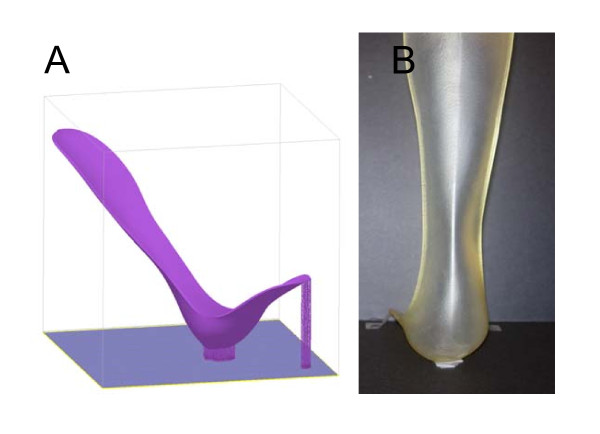
**Rigid RP AFO**. (A) Example of the build platform. (B) Completed rigid RP AFO prototype.

The prototype built using the Acura 40 resin is shown in Figure 6. The model had to be built in an inclined orientation since it did not fit sideways (Figure 6A). The build cycle consisted of 2,269 layers of resin and was built in the total time of 16.7 hours due to the large z-build dimension. Fitting of the rigid RP AFO prototype was excellent.

**Figure 7:**
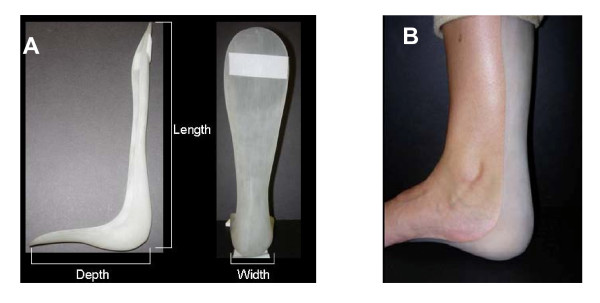
**Flexible RP AFO**. A) The flexible RP AFO. (B) The positioning and fitting of the flexible RP AFO to the leg of the subject.

A second prototype was built from the same STL model file but using a more flexible SOMOS 9120 resin. The dimensions of the final prototype AFO and the prefabricated AFO were very closely matched. The weight of the flexible RP AFO was lower by 21%. Figure 7A shows the flexible RP AFO. Figure 7B shows the flexible RP AFO being worn by the subject recruited for the scanning. The optimal fit of the AFO geometry to the human subject anatomy was evident from visual inspection and the subject expressed great comfort whilst wearing it.

### Testing and Validation

Analysis of the spatio-temporal gait parameters showed that the subject walked very consistently across the four testing conditions. Differences between the conditions based on the range (minimum and maximum values) of each parameter for the left and right leg were less than 10%. When comparing only the right side, on which the AFOs were worn, the differences between conditions for each of the parameters reduced to 5% or less (Table 2). This indicates that observed changes in the kinematics and kinetics of gait are likely due to differences in the properties and behavior of the AFOs rather than to fluctuations in speed or step length of the subject during the walking trials for each condition.

**Table 2:** Mean (± SD) spatiotemporal gait parameters of the right side for the 4 testing conditions

Parameter	No AFO	Standard AFO	Flexible RP AFO	Rigid RP AFO
**Walking speed (m/s)**	1.49 ± 0.05	1.46 ± 0.02	1.44 ± 0.05	1.50 ± 0.06
**Step length (m)**	0.79 ± 0.02	0.79 ± 0.01	0.79 ± 0.03	0.82 ± 0.03
**Double support time (s)**	0.22 ± 0.02	0.24 ± 0.01	0.24 ± 0.01	0.23 ± 0.01

**Figure 8:**
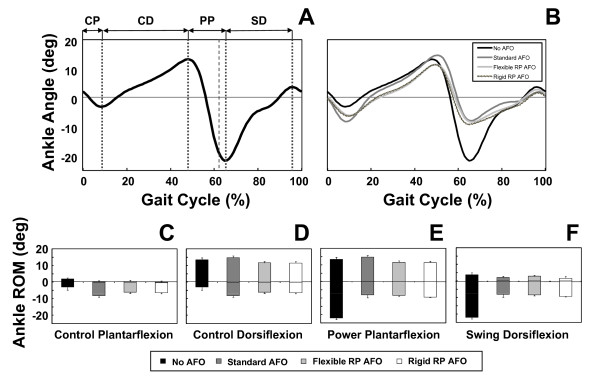
**Ankle kinematics during the 4 testing conditions**. (A) Average profile of ankle plantarflexion-dorsiflexion for five gait cycles of the No AFO condition (i.e. shoes only). The larger dashed vertical line represents the instance of toe-off and the lighter dashed vertical lines separate four different sub-phases of ankle function during the gait cycle (see text for details). (B) Average profiles of ankle plantarflexion-dorsiflexion for five gait cycles of the four testing conditions. Panels C - F show the mean (± SD) range of motion (RoM) in ankle plantarflexion-dorsiflexion for the four sub-phases illustrated in panel A for each of the four AFO conditions.

The ankle kinematics showed the effect of the three tested AFOs. Figure 8A shows the mean plantarflexion-dorsiflexion trajectory of the right ankle for one gait cycle collected during the walking trials performed without AFO. This pattern is typical of individuals without gait abnormalities. For the sake of analyzing the ankle biomechanics, we divided the gait cycle into four sub-phases (see Figure 8A): controlled plantarflexion (CP) after initial contact, controlled dorsiflexion (CD) as the lower leg progresses forward over the foot, power plantarflexion during push-off (PP), and dorsiflexion during swing (SD) to assist foot clearance. The use of an AFO affected the ankle trajectory during these phases (see Figure 8B). Using the above-defined sub-phases, we compared the movement of the right ankle for the four testing conditions (see Figure 8B) to assess the performance of the three AFOs (prefabricated AFO, Flexible RP AFO, and Rigid RP AFO) and compare the observed kinematic trajectories with the data gathered without using an AFO.

Figure 8B shows that the ankle is slightly more plantarflexed at initial contact when wearing no AFO compared to wearing an AFO, and that for each of the AFO conditions initial contact was made with the ankle-foot complex in a more neutral position. This is likely due to the AFOs being made from castings and scans, respectively, of the subject's foot set in a neutral position. During controlled plantarflexion (CD) the ankle showed a similar range of motion (RoM) for each of the AFOs with the standard, prefabricated AFO allowing slightly more plantarflexion compared to the RP AFOs (Figure 8C). This may be due to greater compliance of the polypropylene material from which the standard AFO was made.

During the phase of controlled dorsiflexion (CD), the standard AFO allowed more RoM compared to the two RP AFOs, which performed similarly (Figure 8D). This greater RoM was due to a combination of greater plantarflexion during the CP phase and also greater dorsiflexion during the CD phase.

The ankle showed the greatest RoM during the power plantarflexion (PP) phase at push-off when the subject was wearing no brace since the movement of the ankle was not restricted by an AFO. When wearing the AFOs, the amount of plantarflexion was substantially decreased (Figure 8B) while the RoM during the PP phase was slightly greater for the standard AFO compared to the two RP AFOs (Figure 8E).

In the final phase of dorsiflexion during swing (SD), the ankle showed the greatest RoM when it was not restricted by an AFO, while the three AFO testing conditions showed lower but similar ranges of motion (Figure 8F). This was partly due to the reduced amount of plantarflexion achieved during the PP phase. Importantly the two RP AFOs enabled a similar amount of ankle dorsiflexion at the end of swing as that allowed by the standard AFO (Figure 8B).

**Figure 9:**
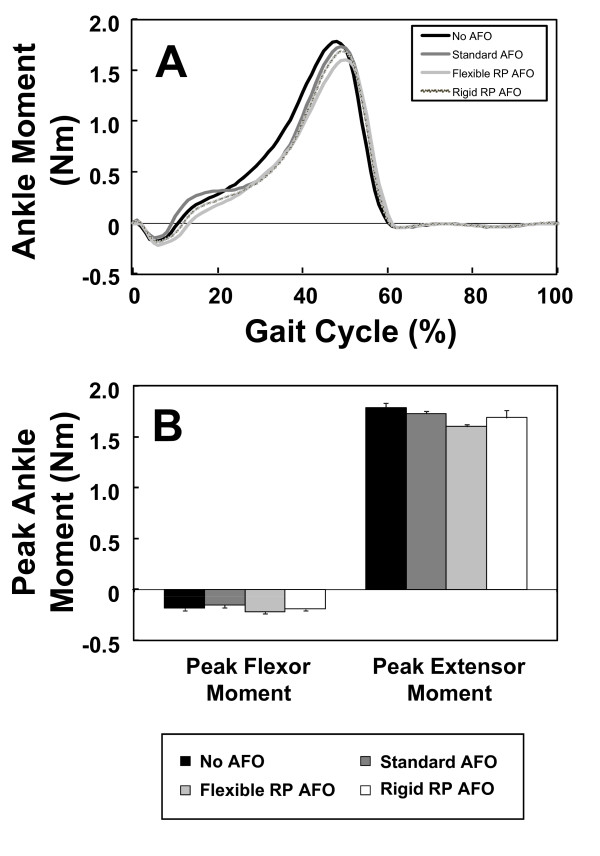
**Ankle kinetics during the 4 testing conditions**. (A) Average profiles of ankle flexor-extensor moments for five gait cycles of the four testing conditions. (B) Mean (± SD) peak ankle extensor and flexor moments for the four testing conditions.

**Figure 10:**
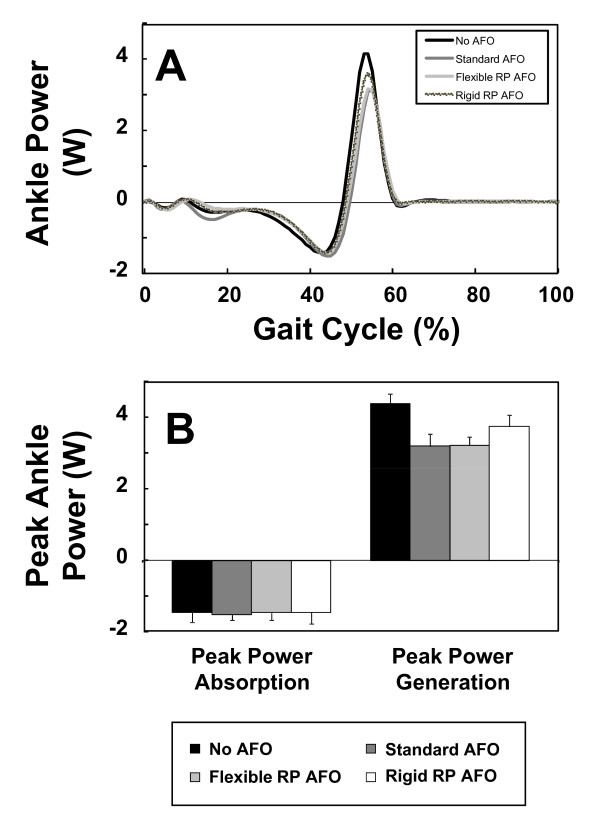
**Ankle power during the 4 testing conditions**. (A) Average profiles of ankle powers for five gait cycles of the four testing conditions. (B) Mean (± SD) peak power absorption and power generation at the ankle for the four testing conditions.

The kinetics of the ankle (joint moments and powers) also revealed that the two RP AFOs performed similarly to the standard AFO. Figure 9A shows the mean right ankle flexion/extension moment during the walking trials for each testing condition. It is evident that the ankle moment profile for the three AFOs was similar. The peak flexor moment for each AFO testing condition was slightly smaller than that for the no-AFO testing condition (Figure 9B). When comparing the profiles of ankle power, we observed similarities across the three AFO testing conditions (Figure 10) with a general reduction in peak power generation compared to the no-AFO condition. This attenuated peak power is likely due to the restricted plantarflexion of the ankle during push off imposed by the AFOs.

Overall, when comparing the three AFOs, it was clear that they performed similarly in terms of controlling ankle kinematics and kinetics during the gait cycle.

The flexible RP AFO performed almost identically to the standard AFO. Both required less ankle power than normal (i.e. with no AFO). The rigid AFO results showed that this testing condition was associated with high ankle power; most likely because the rigid AFO provided resistance to bending that the subject had to overcome. Despite differences among AFO's, it was noted that the change in ankle power was still relatively small, and that increased material flexibility would have been likely to help improving performance.

## Conclusions

In this paper, we presented a process to combine state of the art 3D scanning hardware and software technologies for human surface anatomy with advanced RP techniques so that novel custom made orthoses and rehabilitation devices can be rapidly produced. Two custom-fit AFOs were rapidly prototyped to demonstrate the proposed process. Preliminary biomechanical data from gait analyses of one subject wearing the AFOs indicated that the RP AFOs can match the performance of the standard, prefabricated, polypropylene design. This new platform technology for developing custom-fit RP orthoses has the potential to provide increased freedom with geometric features, cost efficiencies and improved practice service capacities while maintaining very high quality-of-service standards. In the long run, this technology aims at bringing the manufacturing of orthoses from the current manual labor/expert craftsman's skills to a 21^st ^century computerized design process. The proposed technology has the potential for increasing the numbers of patients serviced per year per orthotist while reducing overall the orthosis fabrication cost and time.

## Competing interests

The authors declare that they have no competing interests.

## Authors' contributions

CM, PB, ML: conceived the study and participated in the design and data analysis.

RGR, MLS, AC, KG, LG, SS: carried out the design, fabrication, testing of the RP AFOs.

BLP: participated in the testing of the RP AFOs and performed the data analysis.

JD, RD: participated in the design and data analysis.

All authors read and approved the final manuscript.
